# Supplementary material for: Polar confinement of a macromolecular machine by an SRP-type GTPase
Source: Nat Commun. 2024 Jul 10;15:5797. doi: 10.1038/s41467-024-50274-4 (PMC11236974; doi:10.1038/s41467-024-50274-4)
Supplement: Supplementary file 1 — Supplementary Information [file 41467_2024_50274_MOESM1_ESM.pdf]

Supplementary Information for:

Polar confinement of a macromolecular machine by an SRP-type GTPase

Anita Dornes[1,#], Lisa Marie Schmidt[2,#], Christopher-Nils Mais[1], John C. Hook[2], Jan Pané-Farré[1], Dieter Kressler[3], Kai Thormann[2*], and Gert Bange[1,4*]

[1]Philipps-University Marburg, Center for synthetic Microbiology (SYNMIKRO) and Department of Chemistry, Hans-Meerwein-Strasse 6, C07, 35043 Marburg, Germany
[2]Justus-Liebig-Universität, Department of Microbiology and Molecular Biology, Heinrich-Buff-Ring 26, 35392 Giessen, Germany
[3]University of Fribourg, Department of Biology, Chemin du Musée 10, 1700 Fribourg, Switzerland
[4]Max-Planck-Institute for terrestrial Microbiology, Molecular Physiology of Microbes, Karl-von-Frisch Strasse 14, 35043 Marburg, Germany

[#]These authors contributed equally to this work.
*Correspondence:
gert.bange@synmikro.uni-marburg.de
kai.thormann@mikro.bio.uni-giessen.de

The file contains:
Supplementary Figures 1 – 5
Supplementary Tables 1 – 6

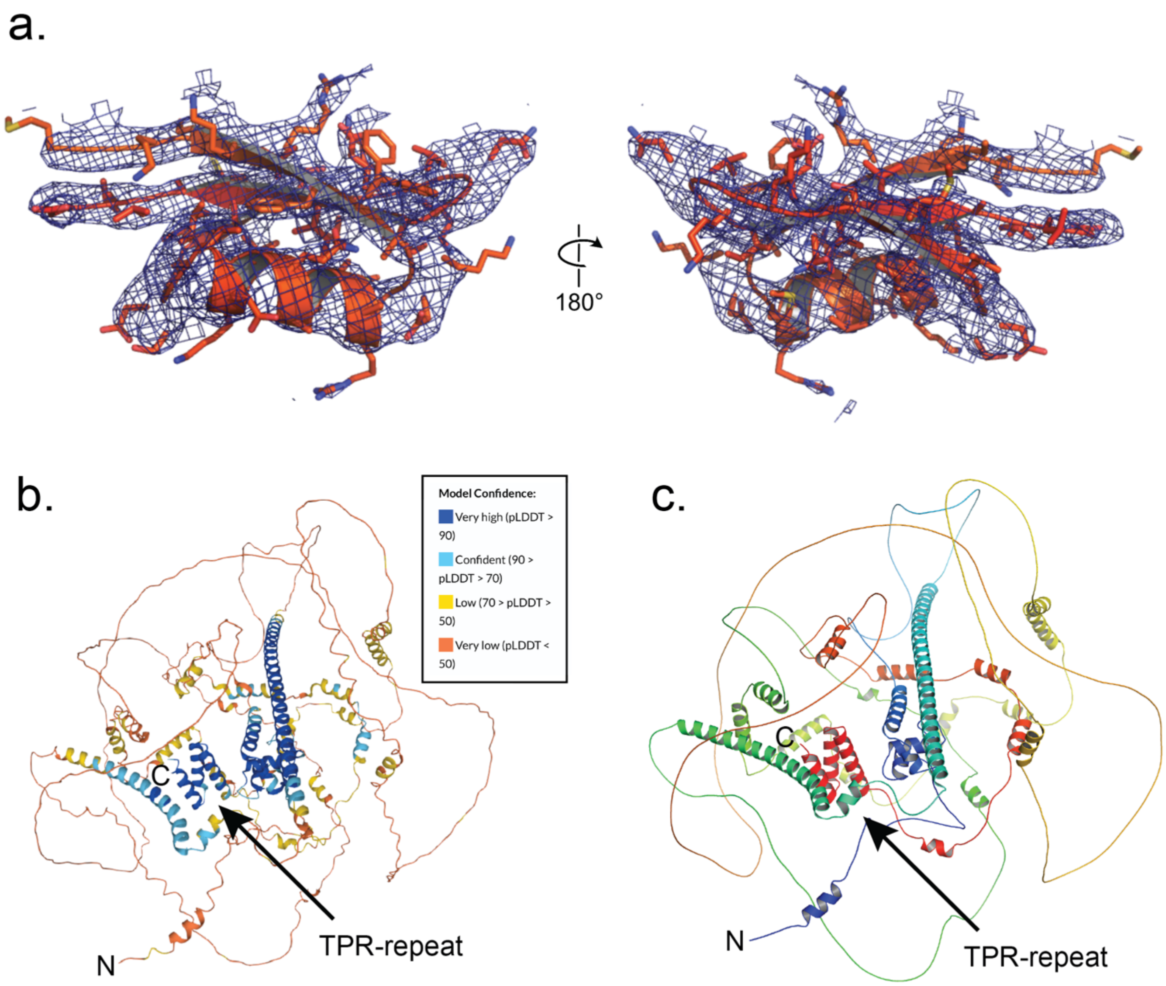

Supplementary Figure 1. a. Electron density of FID domain of *Sp*FlhF. The $2F_{obs}$-F_{calc} electron density map after final refinement is shown as a blue mesh at 1σ. Protein is represented as cartoon with side chains as sticks. **b.-c. Alpha-Fold (1) model of HubP. b. Overall Confidence Assessment.** The confidence analysis of the HubP model reveals a predominantly low level of confidence. The accompanying inset provides a color-coded reference corresponding to the confidence scores. **c.** The Alpha-Fold model of HubP is presented in a simplified cartoon format, employing a rainbow color scheme to depict the sequence from N- to C-terminus. Notably, 'N' and 'C' signify the N-terminus and C-terminus, respectively. The code of the model is: AF-A4Y880-F1 (last assessed in January 2024).

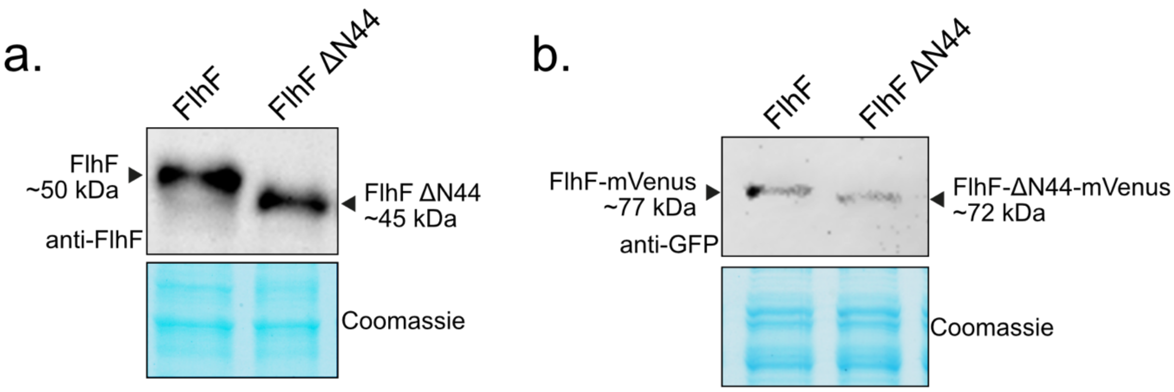

Supplementary Figure 2. The deletion constructs of the 44 aa N-terminal domain of FlhF are stable produced in *Shewanella*. Protein extracts from the respective *Shewanella* strains were separated by SDS-PAGE, transferred to membranes and the proteins were visualized by western blotting with antibodies against (**a.**) FlhF or (**b.**) GFP. For each strain the same OD units (10) were loaded for each sample.

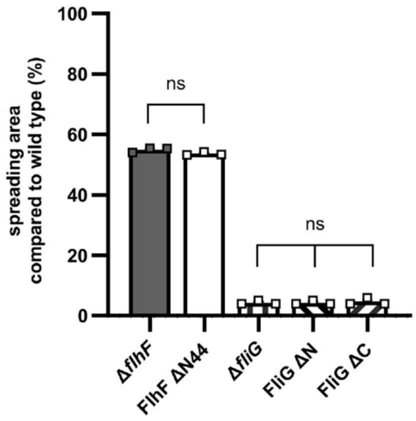

Supplementary Figure 3. Spreading behavior of *S. putrefaciens* is impaired by deletion of the N- or C-terminal domain of FliG or the 44 aa N-terminal domain of FlhF. Strains that are directly compared with each other were spotted on the same plate. Data from three independent experiments and the corresponding error bars are shown. A two-way ANOVA was performed (ns = not significant).

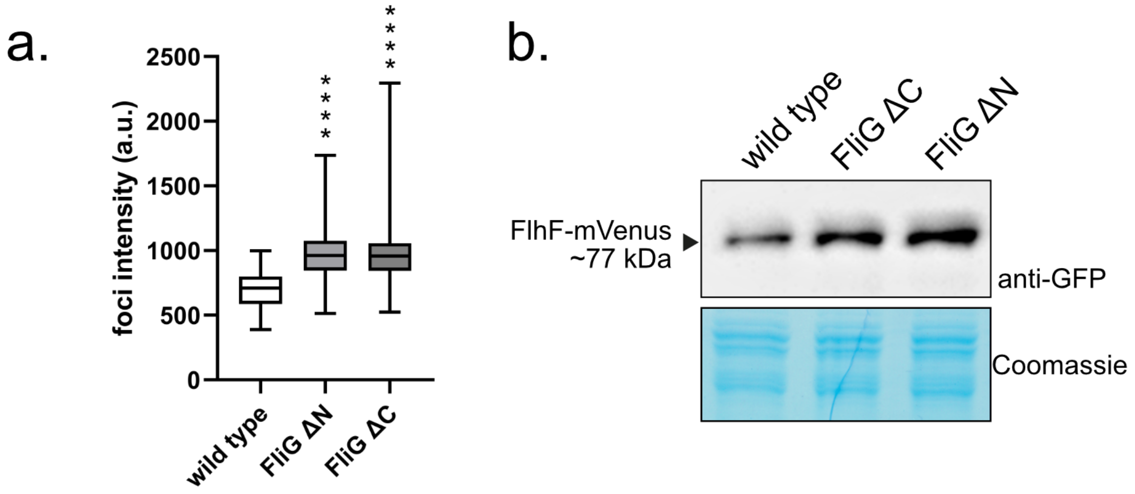

Supplementary Figure 4. The deletion of the N- or C-terminal domain of FliG results in a FlhF-mVenus accumulation. a. Quantification of FlhF-mVenus foci intensity (a.u. arbitrary units). Asterisks represent a p-value <0.0001 (Two-way ANOVA). **b.** Protein levels of FlhF-mVenus. Protein extracts from the respective *Shewanella* strains were separated by SDS-PAGE, transferred to membranes and the proteins were visualized by western blotting with antibodies against GFP.

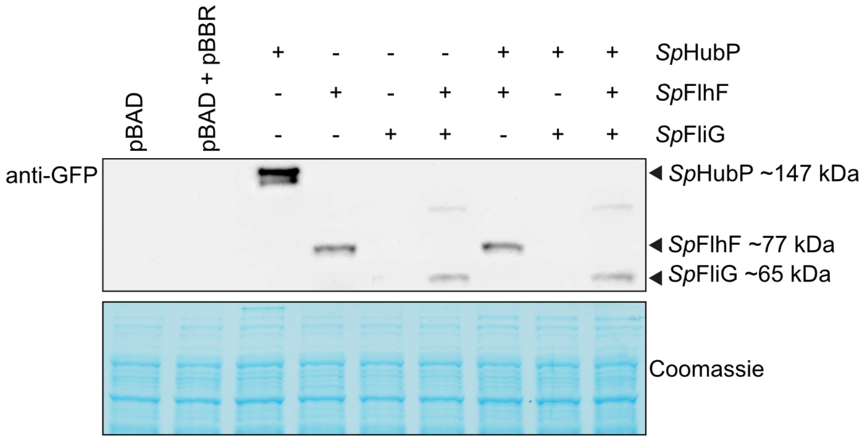

Supplementary Figure 5. The *Shewanella* proteins are stable produced in *E. coli* DH5α. Protein extracts from the respective *E. coli* strains were separated by SDS-PAGE, transferred to membranes and the *Shewanella* proteins HubP (*Sp*HubP), FlhF(*Sp*FlhF) or FliG (*Sp*FliG) were visualized by western blotting with antibodies against GFP. For each strain the same OD units (10) were loaded for each sample. pBAD and pBBR are loaded as empty vector controls.

Supplementary Table S1. Data collection and refinement statistics for the structure determination of the FlhF-FID domain.

**Data collection**	
Space group	$P4_3$
Cell dimensions	
a, *b*, *c* (Å)	106.53 106.53 106.62
α, β, γ (°)	90 90 90
Wavelength (Å)	0.976990
Resolution (Å)	37.68 - 3.01 (3.118 - 3.01)
R_{merge}	0.07745 (2.6)
I / σI	19.39 (0.90)
Completeness (%)	99.72 (99.83)
Redundancy	13.5 (14.0)
CC1/2	0.999 (0.485)
**Refinement**	
Resolution (Å)	37.68 - 3.01 (3.118 - 3.01)
No. reflections	23664 (2371)
R_{work} / R_{free}	0.24/0.27
No. atoms	4269
Protein	4269
Ligand/ion	0
Water	0
B-factors	147.82
R.m.s. deviations	
Bond lengths (Å)	0.006
Bond angles (°)	0.76
Ramachandran	
Favored (%)	94.96
Allowed (%)	5.04
Outliers (%)	0.00

Supplementary Table 2. *Escherichia coli* strains used in this study.

Strain	Genotype	Reference
**DH5α*λpir***	*sup E44, ΔlacU169 (ΦlacZΔM15), recA1, endA1, hsdR17, thi-1, gyrA96, relA1, λpirphage lysogen*	(2)
**WM3064**	*thrB1004 pro thi rpsL hsdS lacZ ΔM15 RP4-1360 Δ(araBAD) 567ΔdapA 1341::[erm pir(wt)]*	W. Metcalf, University of Illinois, Urbana-Champaign

Supplementary Table 3. *Shewanella putrefaciens* CN-32 strains used in this study.

Strain	Genotype	Reference
**wild type**	wild type strain of *S. putrefaciens* CN-32	(3)
**Δ*flhF***	deletion of the gene *flhF* (Sputcn32_2561)	(4)
**Δ*fliG***	deletion of the gene *fliG* (Sputcn32_2575)	This study
***flgE1* T183C**	markerless in-frame substitution of Thr183 to Cys in the polar hook protein FlgE1 (Sputcn32_2594)	(5)
flgE1* T183C Δ*flhF	markerless in-frame substitution of Thr183 to Cys in the polar hook protein FlgE1 (Sputcn32_2594), deletion of the gene *flhF* (Sputcn32_2561)	This study
***flgE1* T183C FlhF ΔN44**	markerless in-frame substitution of Thr183 to Cys in the polar hook protein FlgE1 (Sputcn32_2594) and deletion of first N-terminal 44 amino acids of the gene flhF (Sputcn32_2561)	This study
**FlhF-1xGS-mVenus**	C-terminal mVenus tag of FlhF (Sputcn32_2561) linked with Gly and Ser	(6)
**FlhF ΔN44-1xGS-mVenus**	C-terminal mVenus tag of FlhF without first N-terminal 44 amino acids (Sputcn32_2561) linked with Gly and Ser	This study
**FlhF ΔN44**	Deletion of first N-terminal 44 amino acids of the gene *flhF* (Sputcn32_2561)	This study
***$flaB_1$* T166C *$flaA_1$* T174C $FliG_1$ ΔN**	markerless in-frame substitution of Thr166 to Cys in the polar major flagellin protein FlaB1 (Sputcn32_2585) and Thr174 to Cys in the polar minor flagellin protein FlaA1 (Sputcn32_2586), deletion of the N-terminal domain (Δ2-85) of FliG(Sputcn32_2575)	This study
**FlhF-1xGS-mVenus $FliG_1$ ΔN**	C-terminal mVenus tag of FlhF (Sputcn32_2561) linked with Gly and Ser, deletion of the N-terminal domain (Δ2-85) of FliG(Sputcn32_2575)	This study
***$flaB_1$* T166C *$flaA_1$* T174C $FliG_1$ ΔC**	markerless in-frame substitution of Thr166 to Cys in the polar major flagellin protein FlaB1 (Sputcn32_2585) and Thr174 to Cys in the polar minor flagellin protein FlaA1 (Sputcn32_2586),deletion of the C-terminal domain (Δ209-348) of FliG(Sputcn32_2575)	This study
**FlhF-1xGS-mVenus $FliG_1$ ΔC**	C-terminal mVenus tag of FlhF (Sputcn32_2561) linked with Gly and Ser, deletion of the C-terminal domain (Δ209-348) of FliG (Sputcn32_2575).	This study

Supplementary Table 4. Plasmids used in this study.

General plasmids:

Plasmid	Description	Reference
**pNPTS138-R6KT**	mobRP4+ ori-R6K *sacB;* β-galactosidase fragment alpha; suicide vector for *in-frame* deletions or integrations in *S. putrefaciens*; Kan^r	(7)
**pBAD33**	Empty vector control	(7)
**pBBR1-MCS2**	Empty vector control	(7)

***Escherichia coli* plasmids:**

Plasmid	Description	Reference
**pBBR1-MCS2_HubP**	Expression of HubP (Sputcn32_2442)	This study
**pBAD33_HubP-GGS-sfGFP**	Expression of HubP (Sputcn32_2442) fused to GFP with Gly, Gly and Ser	This study
**pBAD33_FlhF_mVenus-GSGGG-FliG**	Expression of FlhF (Sputcn32_2561) and FliG (Sputcn32_2575) fused to mVenus with Gly, Ser, Gly, Gly, and Gly	This study
**pBAD33_mVenus-GSGGG-FliG**	Expression of FliG (Sputcn32_2575) fused to mVenuswith Gly, Ser, Gly, Gly, and Gly	This study
**pBAD33_FlhF-1xGS-mVenus**	Expression of FlhF (Sputcn32_2561) fused to mVenuswith Gly and Ser	This study
**pBAD33_mCherry-2xGGS-FliF_FliG**	Expression of FliG (Sputcn32_2575) and FliF (Sputcn32_2576) fused to mCherry with 2x Gly, Gly and Ser	This study
**pBAD33_FlhF_mCherry-2xGGS-FliF**	Expression of FlhF (Sputcn32_2561) and FliF (Sputcn32_2576) fused to mCherry with 2x Gly, Gly and Ser	This study
**pBAD33_FlhF_mCherry-2xGGS-FliF_FliG**	Expression of FlhF (Sputcn32_2561), FliG (Sputcn32_2575) and FliF (Sputcn32_2576)fused to mCherry with 2x Gly, Gly and Ser	This study
**pBAD33_mCherry-2xGGS-FliF**	Expression of FliF (Sputcn32_2576) fused to mCherrywith 2x Gly, Gly and Ser	This study
**pET24d_FliG1_N-His**	Expression of N-terminal His-tagged polar FliG	This study
**pET24d_FlhF_1-137_N-Strep**	Expression of N-terminal StrepII-tagged FlhF B-domain amino acids 1-137	This study
**pET24d_FlhF_1-60_N-Strep**	Expression of N-terminal StrepII-tagged FlhF B-domain amino acids 1-60	This study
**pET24d_FlhF_10-137_N-Strep**	Expression of N-terminal StrepII-tagged FlhF B-domain amino acids 10-137	This study
**pET24d_FlhF_20-137_N-Strep**	Expression of N-terminal StrepII-tagged FlhF B-domain amino acids 20-137	This study
**pET24d_FlhF_30-137_N-Strep**	Expression of N-terminal StrepII-tagged FlhF B-domain amino acids 30-137	This study
**pET24d_FlhF_1-44_C-His**	Expression of C-terminal His-tagged FlhF B-domain amino acids 1-44	This study
**pET24d_FlhF_C-Strep**	Expression of C-terminal StrepII-tagged FlhF	This study
**pET24d_FliG-MC_N-His**	Expression of N-terminal His-tagged polar FliG-MC domains	This study
**pET24d_FliG-NM_N-His**	Expression of N-terminal His-tagged polar FliG-NM domains	This study
**pGAT3_FliF-C**	Expression of N-terminal GST-tagged polar FliF-C	This study
**pET24d_FliM_N-His**	Expression of N-terminal His-tagged polar FliM	This study

**pET24d_FliN**	Expression of polar FliN	This study
**pET24d_HubP-860-1097_N-Strep**	Expression of N-terminal StrepII-tagged HubP amino acids 860-1097	This study
**pET24d_FlhF_C-His**	Expression of C-terminal His-tagged FlhF	This study
**pET24d_HubP-860-1033_N-Strep**	Expression of N-terminal StrepII-tagged HubP amino acids 860-1097	This study
**pET24d_HubP-1033-1097_N-Strep**	Expression of N-terminal StrepII-tagged HubP amino acids 860-1097	This study
**pET24d_FlhF-NG_C-His**	Expression of C-terminal His-tagged FlhF-NG	This study
**pET24d_FlhG_N-His**	Expression of N-terminal His-tagged FlhG	This study

***Shewanella putrefaciens* CN-32 plasmids:**

Plasmid	Description	Reference
**pNPTS138-R6KT FlhF-1xGS-Venus**	in frame complementation of *flhF* (Sputcn32_2561) with FlhF-1xGS-mVenus; Kan^r	(6)
**pNPTS138-R6KT *flhF* KO**	deletion of the *flhF* gene (Sputcn32_2561); Kan^r	(4)
**pNPTS138-R6KT *fliG* KO**	deletion of the *fliG* gene (Sputcn32_2575); Kan^r	This study
**pNPTS138-R6KT FlhF ΔN44**	Deletion of first 44 N-terminal amino acids in *flhF* (Sputcn32_2561); Kan^r	This study
**pNPTS138-R6KT FlhF ΔN44-GS-mVenus**	Deletion of first 44 N-terminal amino acids in *flhF*fused to mVenus (Sputcn32_2561); Kan^r	This study
**pNPTS138-R6KT FliG ΔN**	deletion of the N-terminal domain (Δ2-85) of FliG(Sputcn32_2575); Kan^r	This study
**pNPTS138-R6KT FliG ΔC**	deletion of the C-terminal domain (Δ209-348) of FliG (Sputcn32_2575); Kan^r	This study

Yeast two-hybrid plasmids:

Plasmid	Description	Reference
**pGBKT7_FlhF**	pGBKT7 plasmid expressing the FlhF bait protein fused to the Gal4 DNA-binding domain	This study
**pGBKT7_FlhF-B**	pGBKT7 plasmid expressing the FlhF B-domain bait protein fused to the Gal4 DNA-binding domain	This study
**pGADT7_FliF-C**	pGADT7 plasmid expressing the FliFc prey protein fused to Gal4 activation domain	This study
**pGADT7_FliG_polar**	pGADT7 plasmid expressing the polar FliG prey protein fused to Gal4 activation domain	This study
**pGADT7_FliG_lateral**	pGADT7 plasmid expressing the lateral FliG prey protein fused to Gal4 activation domain	This study
**pGADT7_FlhG**	pGADT7 plasmid expressing the FlhG prey protein fused to Gal4 activation domain	This study
**pGADT7_FliM**	pGADT7 plasmid expressing the FliM prey protein fused to Gal4 activation domain	This study
**pGADT7_FliN**	pGADT7 plasmid expressing the FliN prey protein fused to Gal4 activation domain	This study
**pGADT7_FlhF**	pGADT7 plasmid expressing the FlhF prey protein fused to Gal4 activation domain	This study

Supplementary Table 5. Oligonucleotides used in this study. The abbreviations are: for: forward; rev: reverse; KO: knock-out (deletion) primer; KI: knock-in (integration) primer, CP: check primer, SP: sequencing primer, PC: plasmid construction

Oligonucleotides used for *Shewanella putrefaciens* CN-32 work:

Name	Sequence	Purpose
**M13**	TGTAAAACGACGGCCAGTCC	CP/SP
**M13r**	CACACAGGAAACAGCTATGACC	CP/SP
**EcoRV-FlhFdN44-KO-for**	CAAGCTTCTCTGCAGGATAACGCGGCTAATTTCGCAGCA	KO
**OL-FlhFdN44-KO-rev**	GTAGAGGACGCATAAGTGGACTACGATGAGCCAAAAGCGAAGG	KO
**OL-FlhFdN44-KO-for**	TTTTGGCTCATCGTAGTCCACTTATGCGTCCTCTACTGGCCG	KO
**EcoRV-FlhFdN44-KO-rev**	GAATTCGTGGATCCAGATGGTAGTGTCGTAAAAGTGATGCAAAACCTATTAAATG	KO
**Check-FlhF-KO-for**	GCATCAGTCAATGCAAGCAACC	CP
**Check-FlhF-KO-rev**	GCACGGATTAATCCAGCATGCT	CP
**EcoRV-flhF KO-fwd**	GCCAAGCTTCTCTGCAGGATGCATAGGCGTCGGTGATTGAGG	KO
**OL-flhF KO-rev**	TAAGTGAAGGCATTTGAGTAGAGTTATGACCCTGG	KO
**OL-flhF KO-fwd**	CTCAAATGCCTTCACTTATGCGTCCTCTACTGG	KO
**EcoRV-flhF KO-rev**	GCGAATTCGTGGATCCAGATGCTAAGCATTCTCCTAAGCTTGTTG	KO
**PstI-fliG1-KO-fwd**	CCCTGCAGATGAAAATGGCCAAGTG	KO
**OL-fliG1-KO-rev**	TTAGAGGAACTCAGCCATTGTCTTGTAACCA	KO
**OL-fliG1-KO-fwd**	ATGGCTGAGTTCCTCTAATCCGAGTGACAATAA	KO
**PspOMI-fliG1-KO-rev**	TCCGGGCCCGATTCAATACCAAGACGTAAAGCG	KO
**Check-fliG1-KO-fwd**	ATTAGTGTTTCAGTTGCTGTGGAC	CP
**Check-fliG1-KO-rev**	AGGATAACGTCATCAGGGTGCA	CP
**EcoRV-FliG1 dN-fwd**	GCGAATTCGTGGATCCAGATGCTGCAGATGAAAATGGCCAAG	KO
**OL-FliG1 dN-rev**	GTTAAAACCCATTCAGCCATTGTCTTGTAACCAG	KO
**OL-FliG1 dN-fwd**	GGCTGAATGGGTTTTAACAGTGAAGAATTCGTTCG	KO

**EcoRV-FliG1 dN-rev**	GCCAAGCTTCTCTGCAGGATCGGTCATCCACATCGATAAGGT	KO
**EcoRV-FliG1 dC-fwd**	GCGAATTCGTGGATCCAGATGCCTCTATCCTCAAGCACTTAG	KO
**OL-FliG1 dC-rev**	ACTCATTTATTTCGCTGCTTGTGCGCCACCT	KO
**OL-FliG1 dC-fwd**	GCAGCGAAATAAATGAGTTCCTCTAATCCGAGTGAC	KO
**EcoRV FLAG-FliG1 C rv**	GCCAAGCTTCTCTGCAGGATATTCAATACCAAGACGTAAAGCGG	KO
**Check-FliG1 dN-fwd**	GCAGTAGGATTCAGCACACAAC	CP
**Check-FliG1 dN-rev**	GCTTCTACCGTGACTGGTTTGA	CP
**EcoRV FlhF C-term fwd**	GCGAATTCGTGGATCCAGATGCAAGAAATGGTTGGACAGCCT	KI
**OL-FlhF-Venus rev**	CACGCTGCCCTCAAATGCACAGGCCATATTATCTG	KI
**OL_Venus fwd**	GCATTTGAGGGCAGCGTGAGCAAGGGCGAGGAGCTGTT	KI
**OL_Venus rev**	GTCATAACTTTACTTGTACAGCTCGTCCATGCC	KI
**OL-FlhF-Venus fwd**	TACAAGTAAAGTTATGACCCTGGATCAAGCAAG	KI
**EcoRV FlhF C-term rev**	GCCAAGCTTCTCTGCAGGATGCCACATCTAAAAATCGGTCGG	KI

Oligonucleotides used for *Escherichia coli* work:

**Name**	**Sequence**	**Purpose**
**pBAD33 for**	GCACGGCGTCACACTTTGCTATG	CP/SP
**pBAD33 rev**	GCCAGGCAAATTCTGTTTTATCAG	CP/SP
**pBBR1 for**	ACCATGATTACGCCAAGCGCG	CP/SP
**pBBR1 rev**	CGTTGTAAAACGACGGCCAGTG	CP/SP
**pBAD-OL-HubP-for**	AATTCGAGCTCGGTACCCAGGAGGGCAAATATGAAATTTCGCACTTCGTATCTTGTCGGT	PC
**OL-HubP-rev**	TCCTTTGCTGCTACCGCCACTAATCTCTTTTAGTAAACGTCCGGCCTC	PC
**HubP-gfp-for**	GAGATTAGTGGCGGTAGCAGCAAAGGAGAAGAACTTTTCACTGGAG	PC
**HubP-gfp-pBAD-rev**	CGACTCTAGAGGATCCCCTTAGGATCCTTTGTAGAGCTCATCCATGCC	PC
**Seq-HubP-1**	GCACAATCCGCTGATGTTGTTTC	SP
**Seq-HubP-2**	GATATTGCAGTAGCCGATACCGAG	SP
**Seq-HubP-3**	ACGGGCTAAATCCAGTTTGGC	SP
**pBBR-HubP-for**	ATCGAATTCCTGCAGCCCAGGAGGGCAAATATGAAATTTCGCACTTCGTATCTTGTCGG	PC

**pBBR-HubP-rev**	AGAACTAGTGGATCCCCCTTAACTAATCTCTTTTAGTAAACGTCCGGCCTC	PC
**Seq-FlhF-1**	GGCTCAGATGCCGTTATCATGT	SP
**Seq-FlhF-2**	ATCGCATTGGCGCCTATGAGC	SP
**pBAD-OL-FlhF-for**	AATTCGAGCTCGGTACCCAGGAGGGCAAATATGAAGATTAAACGATTTTTTGCCAAAGACATGCG	PC
**OL-FlhF-rev**	GCCCTTGCTCACGCTGCCCTCAAATGCACAGGCCATATTATCTGACC	PC
**FlhF-mVenus-for**	TGTGCATTTGAGGGCAGCGTGAGCAAGGGCGAGGAGC	PC
**FlhF-mVenus-pBAD-rev**	CGACTCTAGAGGATCCCCTTACTTGTACAGCTCGTCCATGCCG	PC
**Seq-mVenus-1**	CGTCTATATCACCGCCGACAAGC	SP
**FlhF-FliG-rev**	CACCATATTTGCCCTCCTCTACTCAAATGCACAGGCCATATTATCTGAC	PC
**mVenus-FliG-for**	TAGAGGAGGGCAAATATGGTGAGCAAGGGCGAGGAGC	PC
**mVenus-FliG-rev**	CATGCCGCCGCCGCTGCCCTTGTACAGCTCGTCCATGCCG	PC
**FliG-linker-for**	AAGGGCAGCGGCGGCGGCATGGCTGAGAATAAAACAAAAGAAGCCGC	PC
**FliG-linker-rev**	CGACTCTAGAGGATCCCCTTAGAGGAACTCATCGCCACCACC	PC
**mVenus-FliG-for2**	AATTCGAGCTCGGTACCCAGGAGGGCAAATATGGTGAGCAAGGGCGAGGAGC	PC
**Seq-FliF-1**	GGCAATCTGCTTAGCCCTAGCC	SP
**Seq-FliF-2**	GGACTTTAAACCCGGTGCTGCA	SP
**Seq-FliF-3**	CTCTTGCTCAGTACGGGCAACG	SP
**Seq-mCherry-1**	GGGTTGGGAAGCAAGTTCAGAACG	SP
**mCherry-FliF-for**	AATTCGAGCTCGGTACCCAGGAGGGCAAATATGGTTTCCAAAGGGGAAGAGGACAATATGGC	PC
**mCherry-FliF-rev**	CGACTCTAGAGGATCCCCTCAGCCATTGTCTTGTAACCAGTTTTTCAC	PC
**FlhF-FliF-rev**	AACCATATTTGCCCTCCTCTACTCAAATGCACAGGCCATATTATCTGACC	PC
**FlhF-mCherry-FliF-for**	TAGAGGAGGGCAAATATGGTTTCCAAAGGGGAAGAGGACAATATGG	PC
**FliF-FliG-mCherry-rev**	GCTACCGCCGCTACCGCCTTTGTATAACTCATCCATACCACCAGTCGAATG	PC
**FliF-FliG-for**	GGCGGTAGCGGCGGTAGCATGATTGTCGGGTCTAATTCCGATTTAGCA	PC

Oligonucleotides used for Yeast-Two Hybrid constructs:

Name	Sequence	Purpose

**FlhF-BspHI-for**	TTAATCATGAGCGTGAAGATTAAACGATTTTTTGCCAAAGAC	PC
**FlhF-BamHI-rev**	TTAAGGATCCTTACTCAAATGCACAGGCC	PC
**FlhF-B-domain-BamHI-rev**	TTAAGGATCCTTACGGAATGTCTCGGCGCTCAG	PC
**FliF-C-NcoI-for**	TTAACCATGGGCCCAATGCTAAAACGCCTTATTTATCC	PC
**FliF-C-BamHI-rev**	TTTTGGCTCATCGTAGTCCACTTATGCGTCCTCTACTGGCCG	PC
**FliG1-PciI-for**	TTAAACATGTTGATGGCTGAGAATAAAACAAAAGAAGC	PC
**FliG1-BamHI-rev**	TTAAGGATCCTTAGAGGAACTCATCGCCACCAC	PC
**FliG2-NcoI-for**	TTAACCATGGGCGATAATTACGCCCAAGCAGC	PC
**FliG2-BamHI-rev**	TTAAGGATCCTTAGACAACGACCTGCTCTTC	PC
**FlhG-NcoI-for**	TTAACCATGGGAACCCTGGATCAAGC	PC
**FlhG-BglII-rev**	TTAAAGATCTTTATTCACTCGTTTTTTCCTC	PC
**FliM1-PciI-for**	TTAAACATGTGCGTGAGTGATTTATTAAGC	PC
**FliM1-BamHI-rev**	TTAAGGATCCTTACCCTGCATCCAACAACTC	PC
**FliN1-PciI-for**	TTAAACATGTAGCACAGAAGATACGGGC	PC
**FliN1-BamHI-rev**	TTAAGGATCCCGAATTAAAAAGCTTAAATAA	PC

Oligonucleotides used for protein expression constructs:

**Name**	**Sequence**	**Purpose**
**FliG-PciI-His-for**	TTAAACATGTTGCACCATCACCATCACCATATGGCTGAGAATAAAACAAAAG AAGCC	PC
**FliG-XhoI-rev**	TTAACTCGAGTTAGAGGAACTCATCGCCACCAC	PC
**FlhF-Strep-PciI-for**	TTAAGGTCTCCTCGAGTTATTTTTCGAACTGCGGGTGGCTCCAAGCGCTCCC CTCAAATGCACAGGCCATATTATCTGACC	PC
**FlhF-137-EcoRI-rev**	TTAAGAATTCTTACGGAATGTCTCGGCGCT	PC
**FlhF-60-EcoRI-rev**	TTAAGAATTCTTAAGGTGCAAGAGGTGTCGCGGAC	PC
**FlhF-ΔN10-Strep-PciI-for**	TTAAACATGTCTAGCTGGAGCCACCCGCAGTTCGAGAAAGGCTTAATTAACC ATATGCGTGCCGCTCTG	PC
**FlhF-ΔN20-Strep-PciI-for**	TTAAACATGTCTAGCTGGAGCCACCCGCAGTTCGAGAAAGGCTTAATTAACC ATACTCTCGGCTCAGATGCC	PC
**FlhF-ΔN30-Strep-PciI-for**	TTAAACATGTCTAGCTGGAGCCACCCGCAGTTCGAGAAAGGCTTAATTAACC ATAACAAAAAGGTGAATGGCGG	PC
**FlhF-NcoI-for**	TTAAGGTCTCCCATGGGCAAGATTAAACGATTTTTTGCCAAAGAC	PC
**FlhF-44-XhoI-rev**	TTAAGGTCTCCTCGAGAACCGCGGCGACAA	PC
**FlhF-Strep-XhoI-rev**	TTAAGGTCTCCTCGAGTTATTTTTCGAACTGCGGGTGGCTCCAAGCGCTCCC CTCAAATGCACAGGCCATATTATCTGACC	PC
**FliG1-M-His-NcoI-for**	TTAACCATGGGCCACCATCACCATCACCATACGGCAGCATTAGGTG	PC

**FliG1-M-XhoI-rev**	TTAACTCGAGTTAGCCACCCATTTTCGCTGC	PC
**FliF-C-NcoI-for**	TTAACCATGGGCCCAATGCTAAAACGCCTTATTTATCC	PC
**FliF-C-His-XhoI-rev**	TTAACTCGAGTTAATGGTGATGGTGATGGTGGCCATTGTCTTGTAACCAG	PC
**FliM-His-NcoI-for**	TTAACCATGGGCCATCACCATCACCATCACGTGAGTGATTTATTAAGC	PC
**FliM-XhoI-rev**	TTAACTCGAGTTAAATACGATCTTGGGATG	PC
**FliN-PciI-for**	TTAAACATGTAGCACAGAAGATACGGGC	PC
**FliN-BamHI-rev**	TTAAGGATCCCGAATTAAAAAGCTTAAATAA	PC
**HubP-860-Strep-NcoI-for**	TTAACCATGGCTAGCTGGAGCCACCCGCAGTTCGAGAAAAGCGCGGTGCTGGATTGGGATACAGAG	PC
**HubP-XhoI-rev**	TTAACTCGAGTTAACTAATCTCTTTTAGTAAACGTCCG	PC
**FlhF-His-XhoI-rev**	TTAAGTCGACTTAGTGGTGATGGTGATGATGCTCAAATGCACAGGCCATATTATC	PC
**HubP-1032-XhoI-rev**	TTAAGGTCTCCTCGAGTTAATCAATTATAGCGGCATCGC	PC
**HubP-1033-NcoI-for**	TTAAGGTCTCCCATGGGCGGTGCCTTACTTGGCG	PC
**FlhG-NcoI-for**	TTAACCATGGGCCACCATCACCATCACCATACCCTGGATCAAGCAAG	PC
**FlhG-XhoI-rev**	TTAACTCGAGTTATTCACTCGTTTTTTCCTCTT	PC

Supplementary references:

1. A. W. Senior *et al.*, Improved protein structure prediction using potentials from deep learning. *Nature* **577**, 706-710 (2020).
2. C. D. Ratree Platt, Sei-Kyoung Park, Gregory J. Phillips, Genetic system for reversible integration of DNA constructs and lacZ gene fusions into the Escherichia coli chromosome. *Plasmid* **43**, 12-23 (2000).
3. J. M. Z. James K. Fredrickson, David W. Kennedy, Hailang Dong, Tullis C. Onstott, Nancy W. Hinman, Shu-mei Li, Biogenic iron mineralization accompanying the dissimilatory reduction of hydrous ferric oxide by a groundwater bacterium. *Geochimica et Cosmochimica Acta* **62**, 3239-3257 (1998).
4. F. Rossmann *et al.*, The role of FlhF and HubP as polar landmark proteins in Shewanella putrefaciens CN-32. *Mol Microbiol* **98**, 727-742 (2015).
5. F. M. Rossmann *et al.*, The GGDEF Domain of the Phosphodiesterase PdeB in Shewanella putrefaciens Mediates Recruitment by the Polar Landmark Protein HubP. *J Bacteriol* **201** (2019).
6. E. E. Arroyo-Pérez *et al.*, A conserved cell-pole determinant organizes proper polar flagellum formation. *bioRxiv* 10.1101/2023.09.20.558563 (2023).
7. J. Lassak, A. L. Henche, L. Binnenkade, K. M. Thormann, ArcS, the cognate sensor kinase in an atypical Arc system of Shewanella oneidensis MR-1. *Appl Environ Microbiol* **76**, 3263-3274 (2010).
